# Personality Traits and Internet Addiction among Adolescent Students: The Moderating Role of Family Functioning

**DOI:** 10.3390/ijerph21050520

**Published:** 2024-04-23

**Authors:** Ifeoma Juliet Nwufo, Obinna Osita Ike

**Affiliations:** Department of Psychology, Faculty of the Social Sciences, University of Nigeria, Nsukka 410001, Nigeria; nwufo.ifeoma@unn.edu.ng

**Keywords:** personality, family functioning, internet addiction, adolescent

## Abstract

**Objectives:** Internet addiction is a behavioral addiction characterized by excessive and compulsive use of the internet. The risk of internet addiction among adolescents has risen recently due to an increase in technological advancement and globalization. However, previous studies have focused on the precipitating factors triggering the internet addiction without looking at the exogenous factors and boundary conditions, such as family functioning, that can either sustain or weaken such behavior. Thus, the present study aimed to examine the moderating role of family functioning in the relationship between personality traits and internet addiction among adolescents. **Methods:** This study is a cross-sectional study consisting of 3150 adolescent students in the grade/class level of JSS1-SS3 who were assessed with standardized measures of the Big-Five Personality Inventory, Internet Addictive Test, and Family APGAR Index. Pearson correlation was used to explore the bivariate relationships between the demographic variable and other variables of interest, while Hayes regression-based PROCESS macro for SPSS was used to test the Hypotheses. **Results:** (1) Openness to experience, conscientiousness, extraversion, and neuroticism positively correlated with internet addiction, whereas agreeableness was negatively associated with internet addiction. (2) Positive family functioning correlated negatively with internet addiction. (3) Positive family functioning moderated the relationships of extraversion and agreeableness with internet addiction but not on openness to experience, conscientiousness, and neuroticism with internet addiction. **Conclusions:** Positive family functioning correlated negatively with internet addiction among adolescents, suggesting that improving family functioning would be a valuable tactic for reducing adolescents’ susceptibility to personality-related internet addiction.

## 1. Introduction

The advent of modern information and communication technology (ICT) and its accessibility and availability, including computers and mobile phones, have significantly changed how people connect in society. However, excessive internet usage among adolescents has led to internet addiction. Internet addiction (IA) describes a condition in which a person has lost control over internet usage and continues to use it excessively to the point that it has detrimental effects on such a person’s life [1]. Internet addiction, also known as problematic internet use or compulsive internet use, refers to a behavioral disorder characterized by excessive and compulsive internet use that interferes with daily life, work, relationships, and overall well-being. Individuals with internet addiction often experience a loss of control over their internet usage and may continue to use the internet despite negative consequences [2]. Such excessive internet use has many adverse effects, especially among adolescents, which include various degrees of antisocial behavior, mental health issues like depression, maladaptive cognition, social anxiety [1], as well as a variety of impairments like poor coordination, social function, behavioral control, and emotional regulation [3,4].

Furthermore, adolescents are the most susceptible group to using the internet excessively [5]. They are susceptible to the various addictive temptations that the internet presents during their period of adjustment, including excessive connection to the internet, difficulty managing time while using the internet, the feeling of a gloomy world outside the internet, a decrease in social interactions, an increase in loneliness and depression or decreased social connection with “real” people, a sense that life outside of the internet is boring, and isolation from friends and family [6,7,8,9,10]. Excessive internet use has been identified as one of the most damaging consequences, generating a new type of psychiatric condition that causes physical and mental health issues [1]. Although internet addiction was originally proposed for inclusion in DSM-5 [11], it is not recognized yet as a disorder by itself. Since 2013, internet gaming disorder is the only internet-related behavioral addiction (non-substance related) included in the 11th edition of the International Classification of Diseases (ICD-11) as a clinically significant problem and in the classification of mental and behavioral disorders of the American Psychiatric Association (DSM-5) as a condition for further study [12]. However, not minding its heterogeneity, internet addiction does not yet exist as a diagnosis or specific disorder in either ICD-11 or DSM-5 [13].

According to Okwaraji et al. [14], it is understood that individuals’ failure to control how they use the internet may eventually cause psychological and social issues in their daily lives. Invariably, internet addiction among adolescents is a significant concern on a global scale [15]. It affects young people in various countries and can have a range of adverse consequences on their physical and mental health, academic performance, and overall well-being [16]. In line with this, Karacic and Oreskovic [8] and Kawabe et al. [17] asserted that, globally, the prevalence of internet addiction among adolescents has increased from 38% to 90% presently. Reports from past studies [18,19,20,21,22] clearly indicate that the prevalence of internet addiction is on the rise, which should be a source of concern to all and sundry.

In Nigeria, the widespread availability of the internet and digital devices has transformed the way people communicate, work, and entertain themselves. While these technological advancements offer numerous benefits, they have also given rise to concerns related to problematic internet use and addiction [23]. According to a survey in Nigeria, 110 million people utilize the internet with adolescents making up more than 35% of this population [24]. The situation is pathetic with more than 90% of secondary school students spending more than 16 h daily on the internet, which may constitute an abuse [25]. This demonstrates that internet addiction has become a significant health concern in Nigeria [1].

In addition, several factors have been implicated in the risk factor of internet addiction, such as social anxiety [1], depression [26], and loneliness [14]. However, the underlying mechanism of personality has yet to be fully explored as it concerns adolescents and IA. This is important because personality refers to the dynamic organization within a person, involving the psychophysical condition that determines a person’s signature behavior and thought. This portrays an individual’s unique and relatively stable patterns of behavior, thoughts, and feelings. It connotes the relatively stable patterns of the internal and external aspects of a person’s character that would cause an impact on behavior in various situations [27]. Therefore, it can be assumed that certain personality traits (individual characteristics) would be responsible for the unusual use of the internet [2]. The Big Five personality traits is a psychological model that describes five broad dimensions of personality: openness, conscientiousness, extraversion, agreeableness, and neuroticism. These traits are believed to be relatively stable, constant, predictable, and steady across time [27]. However, Costa and McCrae’s [28] Big Five personality model is used to conceptualize personality in this study. These traits are openness to experience, conscientiousness, extroversion, agreeableness, and neuroticism. Studies, e.g., [29,30,31,32], have demonstrated that personality traits have a link to internet vulnerability. For instance, extant studies, e.g., [33,34,35,36], have demonstrated that those with high levels of openness to experience possess intellectual curiosity, creativity, and open-mindedness, which may heighten their susceptibility to internet addiction.

Conversely, individuals with high levels of conscientiousness typically exhibit discipline and goal orientation, decreasing their chance of developing an addiction to the internet. Extroverted individuals may also be at greater risk of internet addiction if they lack sufficient social support from their family or peers. Less agreeable people may also display a greater tendency towards compulsive internet use. At the same time, those with high levels of neuroticism may resort to using the internet as an ineffective coping mechanism for dealing with stress and anxiety. Personality connotes composite innate and learned mental aptitudes, temperaments, and attitudes that direct an individual’s distinctions in thoughts, feelings, and behaviors [37]. These traits are important because a person’s disposition can be a recipe for developing and maintaining antisocial behavioral patterns such as addictive internet use. The conclusion that distinct personality qualities lead people to extraordinary internet usage can thus be drawn [2].

Leveraging on the theoretical model of the I-PACE model of internet addiction, the link between personality and internet addiction would be lucid. The I-PACE (Interaction of Person–Affect–Cognition–Execution) model of internet addiction is a theoretical framework proposed to understand the development and maintenance of problematic internet use. It was introduced by Brand et al. [38] and has gained traction in the field of internet addiction research. The model integrates various psychological and neurobiological factors to provide a comprehensive understanding of how internet addiction emerges and persists among individuals. The I-PACE model asserts that there are several components that contribute to specific internet-use disorders, such as an individual’s core characteristics (e.g., personality, social cognitions, psychopathology, specific motives for engaging in behavior, and biopsychological constitution), subjectively perceived situations (e.g., being exposed to addiction related factors, negative mood, and personal conflicts), and affective responses (e.g., coping styles, internet related expectancies, and gratification) [38]. These components provide a comprehensive framework for understanding the multifaceted nature of internet addiction and highlight the importance of considering individual, affective, cognitive, and behavioral factors in its etiology and treatment. The model further asserts that individuals’ personality dispositions affect their predisposition to internet addiction; therefore, it is important to investigate common and unique relationships between the different personality profiles (Big Five personality), family functioning, and internet addiction for a more nuanced understanding of the endogenous and exogenous interplay. Thus, a call on the exogenous factors (e.g., family functioning) is needed to address the consequences of the interactions between individuals’ core characteristics, different predisposing factors, mediators, and moderators underlying such associations [39].

However, the call on the exogenous factors that can moderate the relationship between personality and internet addiction has been noted in the literature [1,40]. Research has demonstrated that social anxiety [41] and family functioning [42,43] are predictive factors for internet addiction, albeit with mixed results. However, extant studies [44] have evidenced that family dynamics can serve as a protective factor against the risk factor for adolescents’ internet addiction. This is important since a healthy family functioning’s affective component (attachment, cohesiveness, and fusion) mitigates the likelihood of internet addiction [45]. Conversely, adolescents who lack family ties find comfort in online interaction and are more likely to develop an internet addiction. The ability of healthy, functioning families to properly moderate the numerous developmental hazards that adolescents face and lower the likelihood of maladjustment, such as internet addiction, is also becoming increasingly clear from research [45]. This has been evidenced by extant studies reporting that healthy family functioning reduces the impact of deviant peer affiliation and delinquency on adolescents [46,47]. This suggests that positive functioning can considerably influence and reduce the adverse effect of internet addiction on the five personality traits of adolescents and that exposure to delinquent peers significantly affects addictive behavior.

## 2. The Present Study

It is worth noting that, while there is ongoing debate regarding the classification of internet addiction as a formal disorder, research into the mechanism and boundary conditions that influence, buffer, or attenuate adolescents’ internet addiction interaction continues to be a significant area of interest in the field of psychology and mental health. These mechanisms and boundary conditions (e.g., family functioning) have not been fully explored in regards to personality traits and internet addiction, particularly among African samples. Most of these previous research studies [1,23,44] dwelt on social cognition, problematic internet use, family functioning, and social anxiety without taking cognizance of personality traits and internet addiction as well as the moderating role of family functioning in such relationships. This has resulted in a paucity of research investigating personality traits as a composite variable based on its five facets using the Big Five model and family functioning among adolescents’ internet addiction, particularly in a neglected context like the African context. However, what is less clear now is how family functioning may serve as a boundary condition in the relationship between the facets of the Big Five personality traits and internet addiction among adolescents in a neglected context like Africa. Thus, the present study tends to fill that gap in the literature by examining the influence of the endogenous factor personality traits (openness to experience, conscientiousness, extroversion, agreeableness, and neuroticism) on IA among adolescent students. This study equally explored the role of the exogenous factor family functioning on IA and its moderating effect on such associations. The researchers, based on theoretical underpins, hypothesize the following:

Hypothesis 1a Extraversion will significantly and positively associate with internet addiction among adolescent students, Hypothesis 1b agreeableness will significantly negatively associate with internet addiction among adolescent students, Hypothesis 1c conscientiousness will significantly and negatively associate with internet addiction among adolescent students, Hypothesis 1d neuroticism will have a significant positive relationship with internet addiction among adolescent students, and Hypothesis 1e openness to experience will have a significant positive relationship with internet addiction among adolescent students;

Hypothesis 2 Adolescent students’ internet addiction will negatively correlate with positive family functioning;

Hypothesis 3a Positive family functioning will moderate the association between extraversion and internet addiction such that the positive association between extraversion and internet addiction will be low rather than high for adolescents with positive family functioning, Hypothesis 3b positive family functioning will moderate the association between agreeableness and internet addiction such that the negative association between agreeableness and internet addiction will be high rather than low for adolescents with positive family functioning, Hypothesis 3c positive family functioning will moderate the association between conscientiousness and internet addiction such that the negative association between conscientiousness and internet addiction will be high rather than low for adolescents with positive family functioning, Hypothesis 3d positive family functioning will moderate the association between neuroticism and internet addiction such that the positive association between neuroticism and internet addiction will be low rather than high for adolescents with positive family functioning, and Hypothesis 3e positive family functioning will moderate the association between openness to experience and internet addiction such that the positive relationship will be low rather than high for adolescents with high positive family functioning.

## 3. Methods

### 3.1. Participants and Procedures

The cross-sectional study was carried out in Southeast, Nigeria, from August to November. The participants for the study comprised 3150 adolescent students from 15 secondary schools in the Southeast states of Nigeria. The sample size was calculated with a 5% margin error, a 95% confidence interval, and an estimated sampling population of 15,437 enlisted as adolescent students in the school under study; the recommended minimum sample size using the Raosoft online sample calculator [48] (https://www.raosoft.com/samplesize.html (accessed on 1 November 2023) was 375 participants. Thus, the sample size utilized in this study was above the threshold level. A multi-stage sampling technique was utilized to select the 3150 participants. Fifteen schools were employed, and 35 adolescent students were chosen from each class or grade, ranging from class/grade JS 1 to class/grade SS. As a result, 210 adolescent students who satisfied the inclusion requirements were chosen randomly from each stratum in the various schools using a simple random technique. Proportionate equal sampling was utilized to ensure equal representative samples across the class/grades and schools. The eligibility criteria for this study included the following: adolescents ≤17 years old, adolescents in a secondary school, and adolescents with relative access to the internet. The exclusion criteria included the following: adolescents ≥18 years old, adolescents who are dropout students, and adolescents who do not have access to the internet.

The participants age ranged from 11 to 17 years (mean age = 15.45; SD = 2.11). Males represent 55.6% of the sample, while females constitute 44.4%. Their class distribution is JS 1 (14.6%), JS 2 (15.1%), JS 3 (15.7%), SS 1 (20%), SS 2 (17.8%), and SS 3 (16.8%) (See Table 1).

Informed consent was obtained from the adolescent students and their parents/guardians, and the management of the various schools used to conduct the study gave permission. The older adolescent students (>16 years) granted consent on their own, and the parents or guardians of the minor-aged adolescent students (<16 years) gave consent on their behalf. The researchers recruited and trained research assistants (teachers) who helped conduct the study. The study’s objectives were conveyed to the participants with the help of the research assistants (teachers), who also helped distribute and retrieve copies of the questionnaire. Fifteen research assistants (teachers in various schools) assisted in the data collection during regular school hours. The characteristics of the adolescent students were well represented by a mixture of junior and senior students (i.e., class/grade JSS and SS). All participants received the appropriate disclosures regarding the voluntary nature of their involvement and the confidentiality of their data, which was ensured by the absence of any form of identification to lessen the likelihood of error bias. This study was approved by the Ethical Committee Board, Department of Psychology, University of Nigeria, Nsukka with the Approval Code No: D.PSY.UNN/REC/2023-1-IRB00019 on January 2023. All institutional and governmental ethical guidelines for human experimentation, as outlined in the Helsinki Declaration of 1975 and updated in 2000, were adhered to. Each questionnaire took between 15 and 25 min to administer. Three thousand, two hundred (3200) of the three thousand, two hundred and fifty (3250) copies of the questionnaire that were sent were returned, and 50 of those copies included incorrect answers. Fifty (50) copies from the returned three thousand, two hundred copies were discarded due to incorrect filling and missing information. Thus, three thousand, one hundred and fifty (3150) valid copies of the questionnaire were used to analyze the data, producing a response rate of 96.9%.

### 3.2. Measures

***Personality***. This study utilized John and Srivastava’s [49] 44-item Big Five personality inventory scale to measure the five dimensions of personality—openness to experience, conscientiousness, extroversion, agreeableness, and neuroticism. The response format was scored on a 5-point Likert scale ranging from (1) “strongly disagree” to (5) “strongly agree”. John and Srivastava [49] obtained a Cronbach alpha of 0.90 for extroversion and agreeableness; 0.92 for conscientiousness, neuroticism, and openness to experience; and an overall score of 0.75. In the present study, the Cronbach α values were 0.78 for openness to experience, 0.72 for conscientiousness, 0.73 for extraversion, 0.73 for agreeableness, and 0.75 for neuroticism with an overall score of 0.77. The scale has been previously used in a similar study [11].

***Family Functioning.*** Smilkstein’s [50] Family APGAR Index was utilized to measure the adolescent perception of family function on the parameters of adaptability, partnership, growth, affection, and resolution. Examples of item statements include “I am satisfied with the support I receive from my family when something concerns me”. A 3-point Likert scale with the values “Hardly ever” (0) to “Almost always” (2) served as the foundation for the response structure. A higher score indicates that the family is thought to function more effectively. A reliability coefficient of 0.85 was reported by Smilkstein [50]. In the current investigation, the researchers obtained a Cronbach alpha of 0.75.

***Internet Addiction Test.*** The internet addiction test (IAT) scale was used to assess the extent of internet addiction among adolescents. It consists of 20-items that ask questions such as “How often do you find yourself staying online longer than you intended?”. Respondents are asked to rate their answers on a six-point scale from “Does not apply” (0) to “Always” (5) with the highest score indicating a more severe addiction. Young [51] reported a Cronbach’s alpha of 0.93. In the current investigation, the researchers found a reliability coefficient of 0.87. IAT has been utilized in several studies conducted in Nigeria [52].

### 3.3. Statistical Analyses

Before the analysis, the normality of the sample was studied via the Kolmogorov–Smirnov test, which revealed a normal distribution. Pearson correlation was used to determine the covariate relationship between the demographic variables and other variables of interest. The data were examined using the regression-based PROCESS macro for SPSS version 23 because the robust PROCESS macro can measure the impacts of moderation or interaction [53]. In management sciences and psychological research, the Hayes PROCESS is currently the most widely used approach for moderation assessments [1]. In order to analyze interaction effects, this method centers the predictor variables prior to analysis, does regression-based route analysis, and automatically creates product terms. The association between the relationship variable (for example, personality) and the criterion variable (for example, internet addiction) would be either stronger or weaker in the presence of the moderator(s), for example, family functioning, if the interaction of predictor and moderator (product term) was significant.

## 4. Results

Table 1 depicts the demographic data of the participants. Regarding the participants’ demographics, males represent 55.6% of the sample, while females constitute 44.4%. Their grade/class distribution is JS 1 (14.6%), JS 2 (15.1%), JS 3 (15.7%), SS 1 (20%), SS 2 (17.8%), and SS 3 (16.8%).

Table 2 shows that being female is associated with being older (*r* = 0.06, *p* < 0.05), being in a lower class (*r* = −0.09, *p* < 0.01), being less agreeable (*r* = 0.06, *p* < 0.01), and having more internet addiction (*r* = 0.06, *p* < 0.05). Older students tend to have more traits associated with positive well-being, such as extraversion (*r* = 0.61, *p* < 0.01), agreeableness (*r* = 0.11, *p* < 0.001), conscientiousness (*r* = 0.06, *p* < 0.05), and openness to experience (*r* = 0.10, *p* < 0.01), but they also have more internet addiction (*r* = 0.07, *p* < 0.01). Adolescent students in higher classes report more internet addiction (*r* = 0.07, *p* < 0.01). Extraversion was positively associated with internet addiction (*r* = 0.10, *p* < 0.01). In the same vein, conscientiousness correlated positively with internet addiction (*r* = 0.21, *p* < 0.001).

The results in Table 3 show that extraversion (*B* = 0.35; 95% CI [0.18, 0.53]; *p* > 0.001), neuroticism (*B* = 0.33; 95% CI [0.18, 0.47]; *p* < 0.001), conscientiousness (*B* = 0.45; 95% CI [0.33, 0.57], and openness to experience (*B* = 0.20; 95% CI [0.09, 0.31]; *p* < 0.001) were positively and significantly associated with internet addiction. This shows that internet addiction among adolescent students increased by 0.35, 0.33, 0.45, and 0.20 units for every one-unit rise in extraversion, neuroticism, conscientiousness, and openness to experience, respectively. In contrast, agreeableness (*B* = −0.19; 95% CI [−0.32, −0.66]; *p* < 0.01) and positive family functioning (*B* = −0.39; 95% CI [−0.34, −0.74]; *p* < 0.05) were negatively associated with internet addiction. Whilst for every one-unit rise in agreeableness and positive family functioning, internet addiction decreased by −0.19 and −0.39 units, respectively. Thus, Hypotheses 1a, 1b, 1d, 1e, and 2 were supported, while Hypothesis 1c was not supported.

**Figure 1:**
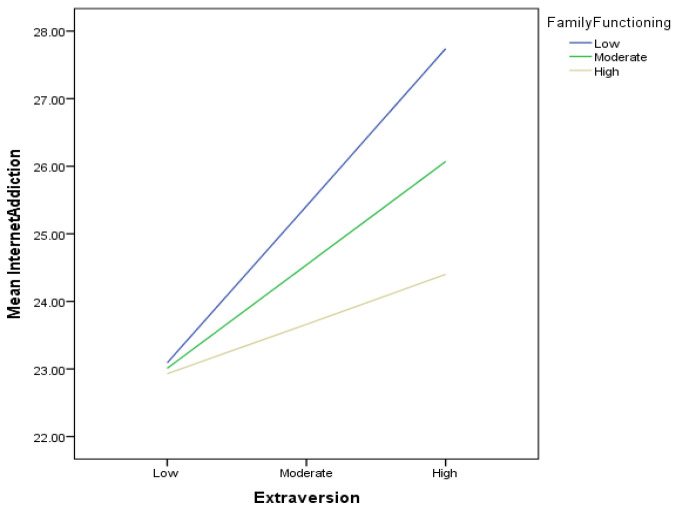
Slope of the moderating role of family functioning on the relationship between extraversion and internet addiction.

Table 4 reveals that the interaction of extraversion and family functioning was significant (*B* = 0.08; 95% CI [0.16, 0.27], *p* < 0.05); thus, Hypothesis 3a was supported, indicating that positive family functioning moderated the relationship between extraversion and internet addiction. The slope of the interaction (Figure 1) indicated that, for those with low family functioning (*B* = 0.54, *t* = 4.11, *p* < 0.001) and those with moderate family functioning (*B* = 0.35, *t* = 4.03, *p* < 0.001), extraversion was associated with more internet addiction. However, for those with high family functioning, extraversion was not significantly associated with internet addiction (*B* = 0.17, *t* = 1.33, *p* = 0.183).

**Figure 2:**
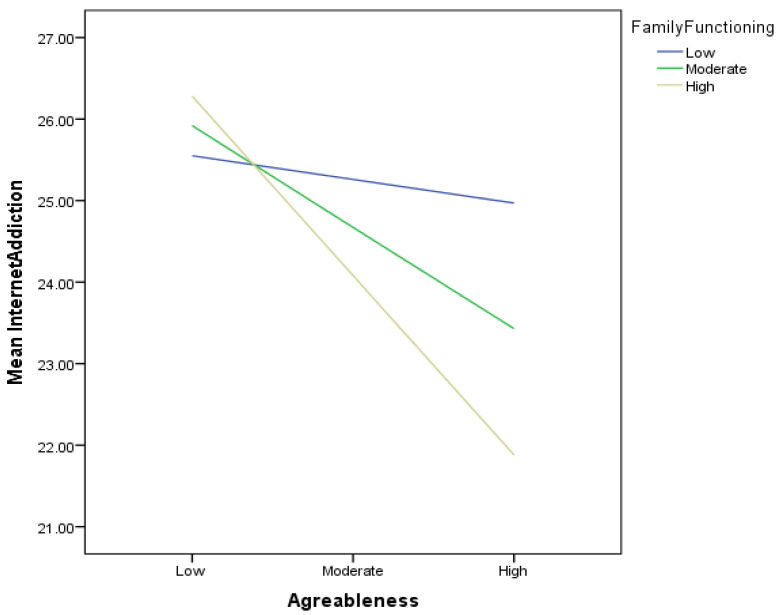
Slope of the moderating role of family functioning on the relationship between agreeableness and internet addiction.

In addition, the interaction of agreeableness and family functioning was also significant (*B* = −0.07; 95% CI [−0.12, −0.01], *p* < 0.05); thus, Hypothesis 3b was supported, indicating that positive family functioning moderated the relationship between agreeableness and internet addiction. The slope of the interaction (Figure 2) indicated that, for those with moderate family functioning (*B* = −0.19, *t* = −2.86, *p* < 0.01) and those with high family functioning (*B* = −3.64, *t* = −3.64, *p* < 0.001), agreeableness was associated with less internet addiction, but for those with low family functioning, agreeableness was not significantly associated with internet addiction (*B* = −0.04, *t* = −0.49, *p* = 0.626). The variables in the equation accounted for 4% *R*^2^ = 0.04, F (6, 1774) = 8.97, *p* < 0.01.

However, positive family functioning did not moderate the relationship between conscientiousness (*B* = −0.03; 95% CI [−0.07, 0.01], *p* = 195), neuroticism (*B* = 0.04; 95% CI [−0.02, 0.11], *p* = 0.181), and openness to experience (*B* = −0.03; 95% CI [−0.08, 0.01], *p* = 0.129) on internet addiction among adolescent students. Thus, Hypotheses 3c, 3d, and 3e were not supported.

## 5. Discussion

The main objective of the current study was to examine the impact of personality traits and family functioning on internet addiction among adolescents. Additionally, this study looked at how family functioning moderated such relationships. According to the current study’s findings, extraversion was significantly and positively associated with internet addiction among adolescent students; thus, Hypothesis 1a was supported. This means that those with traits of extraversion reported high addiction to the internet. This is consistent with the findings of some studies [31,32], which showed a positive association between extraversion and internet addiction. This could be from the inference that extroverts draw energy from interacting with others, which increases their levels of more extensive social networks [54], thereby facilitating online interaction [55], which is a prelude to internet addiction. Thus, adolescents with the trait of extraversion have arrays of social interaction, which increases the proposition of online interaction. Hence, extensive social networks akin to extroverts increases internet addiction via online interaction.

Also, agreeableness was negatively associated with internet addiction; thus, Hypothesis 1b was supported. The result aligns with the findings of past studies [32,35], which showed that agreeableness negatively predicted internet addiction. This is pertinent because individuals who have traits of agreeableness are characterized by virtues of amiableness, kind-hearted, good-natured, reliable, helpful, forgiving, credulous, and honest people. Thus, adolescents who experience such have high-quality prosaic interaction, which protects against internet addiction. Thus, it can be concluded that uncooperative people are drawn to internet addiction because they cannot adapt to their circumstances, portraying that disagreeable people are attracted to internet addiction due to a lack of flexibility and adaptability to their surroundings.

The result also revealed that conscientiousness has a significant positive relationship with internet addiction; thus, Hypothesis 1c was not supported. This contradicts the findings of previous studies [32,33,56], which reported that conscientiousness was negatively associated with internet addiction. This is pertinent because conscientious people excel in delaying gratification, working within the rules, and planning and organizing effectively. This ability, however, makes them not addicted to the internet. However, one plausible reason for the results’ inconsistencies may be technological advancement, where online interaction is now the new normal. For instance, contemporarily, adolescents’ behavioral manifestation is driven by technological patterns of behavior, which can lead to internet addiction. Thus, experiences of such, in most cases, lead to abuse and vulnerability because of social desirability factors, as witnessed in the present study. Equally, certain aspects of conscientious traits and outcomes, such as perfectionism, achievement orientation, and information seeking behavior, can increase the vulnerability of internet addiction among individuals with the conscientious trait. For instance, conscientious individuals often possess a strong curiosity and thirst for knowledge. While this trait can be beneficial, it may also drive them to spend excessive time online seeking information, learning new skills, or engaging in intellectually stimulating activities. Over time, this behavior can escalate into internet addiction if it interferes with other responsibilities or social interactions.

In addition, neuroticism was significantly positively associated with internet addiction; thus, Hypothesis 1d was supported. This is in line with the findings of previous studies [36,56], which showed a positive correlation. Neurotic individuals experience anger, anxiety, irritability, apprehension, depression, and insecurity/vulnerability [57]. This propels them to experience increased stress levels and interpersonal conflicts because they always interpret situations in an alarming, threatening, and harmful direction. Thus, problems in emotion regulation usually influence their ability to think clearly, make decisions, and cope effectively in social interaction. Thus, the effect of such a situation leads to avoidance and solitude with a greater propensity for online interaction [58].

In the same vein, openness to experience was positively correlated with internet addiction, meaning that adolescent students who exhibit openness to experience are more likely to report having a problem with the internet. As a result, Hypothesis 1e was confirmed. Research has discovered that there could be a connection between the level of openness to new experiences among adolescents and their likelihood of experiencing internet addiction. Furthermore, the same research suggests that students with high levels of conscientiousness may have a higher probability of developing internet addiction. Individuals must use technology judiciously and responsibly to avoid any potential negative impact it may have on their physical and mental health in the quest for new ideas. This result is consistent with earlier research findings [32,59] showing that openness to new experiences was a favorable predictor of internet addiction. Thus, individuals who are high in openness to experience are people who like learning, enjoy the arts, and engage in a creative career or hobby [60]. This portrays that adolescent students with openness to experience may experience internet addiction because they like exploring new opportunities in their environment (e.g., the internet) that, if not properly checked, can lead to vulnerability.

Furthermore, positive family functioning was associated negatively with internet addiction; thus, Hypothesis 2a was supported. This coincides with previous findings [1,43], which reported that positive and functional family functioning reduces internet addiction. The finding portrays that positive family functioning increases familial relationships among family members and acts as a protective factor against online interaction due to the fusion and attachment of the family [44]. Lack of familial support and inadequate parental support and involvement are more likely to lead to the development of excessive use of social platforms such as the internet. Hence, online interaction becomes a substitute for offline interaction, where they receive admiration and emotional support [45]. Due to a lack of fusion and familial attachment, dysfunctional family functioning is the risk factor for internet addiction in adolescent students [47].

In addition, positive family functioning moderated the relationship between extraversion and agreeableness on internet addiction but did not on neuroticism, conscientiousness, and openness to experience; thus, Hypotheses 3c and 3d were supported. The result revealed that positive family functioning moderated the relationship between extraversion and internet addiction among adolescent students, indicating that positive family functioning weakens the relationship between extraversion and internet addiction. This aligns with the findings of previous studies [61,62], which showed that positive family functioning is a protective factor for internet addiction. This implies that adolescents’ difficulty with self-control regarding negative social behaviors like internet addiction is counterbalanced by the family’s affective involvement, attachment, and familial fusion.

This study revealed that positive family functioning moderated the relationship between agreeableness and internet addiction. This aligns with previous studies [61,62], which reported that positive family functioning was a protective factor against antisocial behaviors. Thus, the relationship between agreeableness and internet addiction was stronger when family functioning was low.

This indicates that the magnitude of the relationship between agreeableness and internet addiction changes as a function of family functioning [63]. For instance, the agreeableness trait personality and good family functioning are linked to lower levels of online addiction, whereas the agreeableness trait personality and dysfunctional family functioning are linked to higher levels of internet addiction.

However, family functioning did not moderate the relationship between the personality traits of neuroticism, conscientiousness, and openness to experience on internet addiction. A paradigm change in modernity and civilization, where online communication is now the new standard, might lead to a credible explanation. Due to busy schedules at work and the need to address domestic issues, most families spend more time communicating with their kids in person over the phone and online [64]. The aftermath of this in-person or online interaction is a high risk of internet addiction due to neglect in the guidance behavior of parents that are supposed to encourage communication, emotional ties, familial support, and integration [65].

### 5.1. Implications of this Study

This study’s findings have educational and clinical implications. The findings of this study can inform educational policies and practices aimed at promoting healthy internet usage habits among adolescents and reducing the prevalence of internet addiction in school settings. By addressing the interplay between personality traits, family functioning, and internet addiction, educators can implement targeted interventions that support the holistic development and well-being of students. On the clinical implications of this study, the findings highlight the importance of considering personality traits and family functioning in the assessment and treatment of internet addiction among adolescent students. By addressing these factors holistically, clinicians can develop tailored interventions that promote positive outcomes and enhance the well-being of adolescents and their families. This study suggests that family-based intervention (e.g., systemic family therapy) may effectively prevent and reduce adolescent internet addiction by promoting positive family functioning. This will guide the creation of intervention plans and support services (such as social and familial support) for adolescents at risk of developing internet addiction.

In addition, this study’s findings bring to the fore attitudinal factors (personality traits) and contextual and environmental factors (family functioning) in explaining internet addiction among adolescents. This study shows that certain personality traits are linked to internet addiction among adolescents. Thus, addressing these personality traits as part of prevention and intervention programs is crucial as well as the critical role of family functioning in adolescents’ development of internet addiction by emphasizing that family functioning plays a significant role in shaping an adolescent’s personality traits and behaviors.

### 5.2. Limitations and Directions for Future Research

This research has some limitations. First, personality traits and family systems and functioning are dynamic and are susceptible to constant change. Thus, it may be challenging to infer accurate linkage and correlation among these variables of interest in internet addiction, even though control variables were accounted for during the analysis. Future studies should consider using a mixed-method approach to provide a broader spectrum for understanding the multifaceted nature of personality and family systems. Equally, the environmental context concerning cultural diversities should be explored with internet addiction as this could bring to the fore more underlying factors or mechanisms for an apt understanding of this scientific inquiry.

## 6. Conclusions

Personality traits have varied associations (positive and negative) with internet addiction. Positive family functioning is negatively associated with internet addiction among adolescents. Furthermore, positive family functioning moderated the relationship between agreeableness and extroversion such that the negative relationship between agreeableness and internet addiction was strengthened, while the positive relationship between extroversion and internet addiction was weakened among adolescents. From the results, it was found that certain personality traits could be a potential risk factor for internet addiction, which in turn could harm one’s social interactions. This study also revealed that the functioning of one’s family had a significant role in influencing internet addiction. It was observed that positive family functioning acted as a boundary condition for some of the personality traits. Therefore, educating adolescents about the concept of family and the environment in which they grow up can be a protective factor in reducing internet addiction. Thus, addressing not only individual factors such as personality traits but also environmental factors such as importance of positive family functioning, which is a crucial component as an agent of socialization in preventing internet addiction among adolescents, is imperative and sacrosanct.

## Figures and Tables

**Table 1:** Participant characteristics (N = 3150).

Demographic	Frequency	Mean (SD)	%
**Gender**			
**Male**	1750		55.6
**Female**	1400		44.4
**Age (range 11–17 years)**		15.70 (2.17)	
**Class/Grade**			
**JSS 1**	460		14.6
**JSS 2**	476		15.1
**JSS 3**	494		15.7
**SS 1**	630		20.0
**SS 2**	560		17.8
**SS 3**	530		16.8

**Table 2:** Correlations of demographic variables, family functioning, personality traits, and internet addiction.

Variables		1	2	3	4	5	6	7	8	9	10
1	Gender	-									
2	Age	0.06 *	-								
3	Class	−0.09 **	0.61 **	-							
4	Family functioning	−0.03	0.00	0.03	-						
5	Extraversion	−0.04	0.08 **	0.06 *	0.05 *	-					
6	Agreeableness	−0.06 *	0.11 ***	0.15 **	0.21 ***	0.07 **	-				
7	Conscientiousness	0.02	0.06 *	0.06 *	0.12 ***	0.09 **	0.21 ***	-			
8	Neuroticism	−0.01	0.00	−0.01	−0.15 ***	−0.03	−0.27 ***	0.20 ***	-		
9	Openness to experience	−0.01	0.10 **	0.12 **	0.15 ***	0.11 ***	0.43 ***	0.54 ***	−0.05 *	-	
10	Internet addiction	0.09 **	0.13 ***	0.07 **	−0.04	0.10 **	−0.07 **	0.21 ***	0.12 ***	0.10 ***	-

**Note:** *** *p* < 0.001; ** *p* < 0.01; * *p* < 0.05; Gender (0 = boys; 1 = girls).

**Table 3:** Statistical test of significance of the predicting roles of personality traits and family functioning on internet addiction.

Variables	Β	SE	T	95%CL LLCI ULCI
Gender	2.85	0.76	3.77 ***	[1.37, 4.33]
Age	0.83	0.26	3.23 **	[0.33, 1.33]
Class	0.05	0.40	0.12	[−0.72, 0.81]
Extraversion	0.35	0.09	4.03 ***	[0.18, 0.53]
Agreeableness	−0.19	0.07	−2.86 **	[−0.32, −0.66]
Conscientiousness	0.45	0.06	7.86 *	[0.4533, 0.57]
Neuroticism	0.33	0.07	4.43 ***	[0.18, 0.47]
Openness to experience (OE)	0.20	0.06	3.52 ***	[0.09, 0.31]
Family functioning (FF)	−0.39	0.18	−2.19 *	[−0.34, −0.74]

**Note:** * = *p* ≤ 0.05, ** = *p* ≤ 0.01, *** = *p* ≤ 0.001, *β* = Regression Coefficient; SE = Standard Error; *t* = population t value; *p = Probability Level;* LLCI and ULCI = Lower and Upper Limit Confidence Interval; ∆*R*^2^ Adjusted R square; Gender (coded 0 = male, 1 = female).

**Table 4:** Hayes PROCESS Macro results for moderating role of family functioning in association between personality traits and internet addiction.

Variables	Β	SE	T	95%CL LLCI ULCI	∆*R*^2^ ∆*F*
Extraversion X FF	0.08	0.04	1.95 *	[0.16, 0.27]	*R*^2^ = 0.04; *F*(6, 1774) = 9.96 **
Agreeableness X FF	−0.07	0.03	−2.11 *	[−0.12, −0.01]	*R*^2^ = 0.04; *F*(6, 1774) = 8.97 **
Conscientiousness X FF	−0.03	0.02	−1.30	[−0.07, 0.01]	*R*^2^ = 0.07; *F*(6, 1774) = 18.17
Neuroticism X FF	−0.20	0.19	−1.09	[−0.56, 0.16]	*R*^2^ = 0.04; *F*(6, 1774) = 11.48
OE X FF	−0.03	0.02	−1.52	[−0.08, 0.01]	*R*^2^ = 0.04; *F*(6, 1774) = 12.18

**Note:** * = *p* ≤ 0.05, ** = *p* ≤ 0.01, *β* = Regression Coefficient; SE = Standard Error; *t* = population t value; *p = Probability Level;* LLCI and ULCI = Lower and Upper Limit Confident Interval; ∆*R*^2^ Adjusted R square; Gender (coded 0 = male, 1 = female).

## Data Availability

The datasets generated and analyzed during the current study will be available from the corresponding author upon reasonable request.
